# Coated Diammonium Phosphate Combined With Humic Acid Improves Soil Phosphorus Availability and Photosynthesis and the Yield of Maize

**DOI:** 10.3389/fpls.2021.759929

**Published:** 2021-12-16

**Authors:** Qi Chen, Zhaoming Qu, Zeli Li, Zixin Zhang, Guohua Ma, Zhiguang Liu, Yanfeng Wang, Liang Wu, Fuli Fang, Zhanbo Wei, Min Zhang

**Affiliations:** ^1^National Engineering Research Center for Efficient Utilization of Soil and Fertilizer Resources, College of Resources and Environment, Shandong Agricultural University, Tai’an, China; ^2^Key Laboratory of Crop Specific Fertilizer, Ministry of Agriculture, Xinyangfeng Agricultural Technology Co., Ltd., Jingmen, China; ^3^Institute of Applied Ecology, Chinese Academy of Sciences, Shenyang, China

**Keywords:** phosphorus use efficiency, economic benefits, phosphate release, endogenous hormone, phosphatase

## Abstract

Controlled release phosphorus (P) fertilizers and humic acid (HA) applications are two effective and significant techniques or measures for preventing P loss and enhancing maize development. However, the underlying physiological mechanism of how the controlled release P fertilizers combined with HA affect the maize production and P-use efficiency (PUE) remains unknown. The effects of applying coated diammonium phosphate (CDAP) and HA together on soil nutrient supply intensity, soil phosphatase activity, photosynthesis, endogenous hormone contents, and yield of maize, as well as PUE, were examined in this study. In a pot experiment, two types of P fertilizers—CDAP and diammonium phosphate (DAP)– as well as two HA application rates (0 and 45 kg ha^–1^) and two P levels (60 and 75 kg P_2_O_5_ ha^–1^) were utilized. Results showed that the key elements that influence the growth and yield of the maize were the availability of P content in soil, plant photosynthesis, and hormone levels. The combination of CDAP and HA had a greater impact on yield and PUE over the course of 2 years than either DAP alone or DAP combined with HA. Besides, using CDAP in combination with HA increased the yield and PUE by 4.2 and 8.4%, respectively, as compared to the application of CDAP alone at 75 kg P_2_O_5_ ha^–1^. From the twelve-leaf to milk stages, the available P content in the soil was increased by an average of 38.6% with the combination of CDAP and HA compared to the application of CDAP alone at 75 kg P_2_O_5_ ha^–1^. In addition, the application of CDAP combined with HA boosted the activities of ATP synthase, as well as the content of cytokinin (CTK), and hence improved the maize photosynthetic rate (Pn). When compared to the application of CDAP alone or DAP combined with HA, the Pn of CDAP + HA treatments was enhanced by 17.9–35.1% at the same P rate. In conclusion, as an environmentally friendly fertilizer, the combined application of CDAP and HA improved the intensity of the soil nutrient supply, regulated photosynthetic capabilities, and increased the yield and PUE, which is important for agricultural production, P resource conservation, and environmental protection.

## Introduction

Phosphorus (P), as a structural element, is one of the essential macronutrients for plant growth and development (Johnston and Poulton, 2019; Ding et al., 2021). The P deficiency limits virtually all major metabolic processes, in plants, such as photosynthesis and respiration (Plaxton and Tran, 2011). However, agricultural production of over 40% of the world’s arable land is limited by P deficiency (Balemi and Negisho, 2012; Zhu et al., 2018). Therefore, P fertilizers are commonly applied to meet crop demand. Because of sorption, precipitation (usually by interaction with Ca^2+^ and Mg^2+^ in calcareous soils, and Fe^3+^ and Al^3+^ in acidic soils), and microbial immobilization, the P-use efficiency (PUE) of most crops is only 10–15% (Castro and Torrent, 1998; Roberts and Johnston, 2015; Zhu et al., 2018). The applied P accumulates in soils and causes soil degradation and environmental concerns, such as water eutrophication (Leslie et al., 2017). Moreover, the detrimental effects of climate change on P transport in soil and lake eutrophication, such as global warming, drought, and heavy rainfalls, have been exposed (Piao et al., 2010; Fahad et al., 2016, 2020). China is the largest producer and consumer of P fertilizers in the world, but the reserve of phosphate rock, the main source of phosphate in fertilizer, is limited (Zhang et al., 2008; Ma et al., 2011). Therefore, effective P management is of importance for PUE improvement, resource reservation, and environmental protection.

Effective P management involving appropriate P fertilizers is vital for high PUE (Zheng et al., 2016; Tian et al., 2018). Many environmentally friendly methods for increasing P availability have been proposed, including the use of P-solubilizing microorganisms (Adnan et al., 2020; Wahid et al., 2020), partial acidification of rock phosphates (Sarkar et al., 2018), combined application of biochar and P fertilizers (Fahad et al., 2016), and foliar application of P fertilizers (Rafiullah et al., 2020). The application of controlled-release fertilizers is one of these methods. Controlled-release P fertilizers show high PUE in both acidic and alkaline soils. The PUE of controlled-release P fertilizers was reported to be higher than that of water-soluble P fertilizers (Sarkar et al., 2018). Coated diammonium phosphate (CDAP), a controlled-release P fertilizer, releases P according to the demand of the plant, which not only improves PUE and crop yield but also reduces the environmental risk posed by the excessive use of fertilizers (Cruz et al., 2017; Lu et al., 2019). Chen et al. (2020) demonstrated that compared to diammonium phosphate (DAP), CDAP significantly increased maize yield and PUE by 9.65 and 7.72%, respectively.

Humic acid (HA) applied to soil as an activator is also reported to increase the availability of soil P (Zhu et al., 2018). HA consists of aromatic and aliphatic structures harboring various functional groups (mainly oxygen-containing), such as carboxyl (–COOH) and phenolic hydroxyl (–OH). Studies have shown that HA application reduced P fixation, improved the efficiency of low and high solubility P sources, increased P availability, and improved PUE (Çimrin et al., 2010; Rosa et al., 2018; Shafi et al., 2020; Xu et al., 2021). HA improved soil structure by encouraging the formation of stable aggregates, which increased the productivity of soil crops (Zhou et al., 2019). In addition, HA has been shown to improve certain aspects of growth in essential agronomic crops like soybean, wheat, rice, and maize (Calvo et al., 2014; Rosa et al., 2018). As a plant biostimulant, HA increases photosynthesis, reduces transpiration, stimulates root and shoot growth, and enhances stress resistance of plants (Canellas et al., 2013; Dantas et al., 2018; Xu et al., 2021), and is linked with changes in the hormone contents and enzyme activities and enhancement of H^+^-ATPase activity (Zandonadi et al., 2010; Calvo et al., 2014). However, since HA is a weak nutritional material, it cannot supply the nutrient requirements in crop production on its own.

Many research has been conducted on the effects of HA application, the combination of water-soluble P fertilizer and HA, and HA mixed with urea or controlled-release urea on soil quality, plant development, and fertilizer use efficiency (Shafi et al., 2020; Li Z. L. et al., 2021; Xu et al., 2021). Rosa et al. (2018) found that combining HA with phosphate fertilizers (e.g., single superphosphate) boosted root dry matter, and nutrient uptake increased the shoot dry matter output, as compared to biomass produced in soil that had not been treated with HA. To our knowledge, few studies on the effects of combining HA with controlled release P fertilizer on crop production have been conducted. We hypothesized that the combined application of CDAP and HA would improve crop growth, crop yield, and PUE. This study was aimed to: (1) investigate the effects of CDAP combined with HA on soil P availability, (2) understand the roles of photosynthesis and endogenous hormones in the increase of maize production when CDAP and HA are applied together, and (3) determine the factors that influence crop yield and PUE. Findings from this study should give a technological foundation for developing an effective fertilization strategy using controlled-release P fertilizers and biostimulants.

## Materials and Methods

### Soil, Coated Diammonium Phosphate, and Humic Acid Used

The soil for the pot experiment was acquired from a field at the research farm of the National Engineering Laboratory for Efficient Utilization of Soil Fertility Resources (NELEUSFR), Shandong Agricultural University (SDAU), China. It is classified as Typic Hapludalf (Soil Survey Staff, USDA, 1999) or Typic-Hapli-Udic Argosols (Chinese Soil Taxonomy, CRGCST, 2001). Physical and chemical properties of the soil were as follows: pH: 7.83 (1:2.5 soil to water ratio), organic matter content: 12.10 g kg^–1^, total P: 0.32 g kg^–1^, available P: 13.50 mg kg^–1^, NO_3_^––^ N: 71.45 mg kg^–1^, NH_4_^+^-N: 9.45 mg kg^–1^, and available K: 92.32 mg kg^–1^.

The controlled-release P fertilizer, the CDAP (17.2% N, 44.0% P_2_O_5_), was prepared by NELEUSFR, SDAU, China. The coating consisted of 10% of paraffin and 90% of polyurethane. Resin-coated controlled-release urea (43.0% N; 3-month release period) was purchased from Kingenta Ecological Engineering Group Co., Ltd., Shandong, China. The other fertilizers, urea (46% N), DAP (18.0% N, 46.0% P_2_O_5_), and potassium chloride (60.0% K_2_O), were purchased from the local market. The HA (2.0-0%-3.0% N-P_2_O_5_-K_2_O) was purchased from Quanlin Jiayou Fertilizer Co., Ltd., Shandong, China. It had a pH of 5.40 (1:2.5 soil to HA ratio).

### Pot Experiment

The pot experiment was carried out in the research farm of NELEUSFR, SDAU, China. With an average annual temperature of 13.2°C, the experiment location has a moderate continental monsoon climate. The following nine treatments were put up, each with four replications: (1) Control (no P fertilization); (2) P, 100% (DAP at 75 kg P_2_O_5_ ha^–1^); (3) P, 80% (DAP at 60 kg P_2_O_5_ ha^–1^); (4) CP,100% (CDAP at 75 kg P_2_O_5_ ha^–1^); 5) CP, 80% (CDAP at 60 kg P_2_O_5_ ha^–1^); (6) P, 100% + HA (DAP at 75 kg P_2_O_5_ ha^–1^ combined with HA); (7) P, 80% + HA (DAP at 60 kg P_2_O_5_ ha^–1^ combined with HA); (8) CP, 100% + HA (CDAP at 75 kg P_2_O_5_ ha^–1^ combined with HA); and (9) CP, 80% + HA (CDAP at 60 kg P_2_O_5_ ha^–1^ combined with HA).

In each ceramic pot (36.0 cm in height, 30.0 cm in diameter), 1 kg sand was first placed in the bottom to improve aeration and to promote more oxygen supply to the root system (Li Z. L. et al., 2021), and then 20 kg of soil was placed on the top of the sand layer (Yu et al., 2021). Before usage, the test soil was air-dried, blended equally, and sieved. The sand (0.35–0.5 mm) used was purchased from the local market.

For the control treatment, nitrogen and potassium fertilizers were applied once as a basal fertilizer at 225 kg N ha^–1^ and 150 kg K_2_O ha^–1^, respectively, whereas for the other treatments, nitrogen, P, and potassium fertilizers were applied at 225 kg N ha^–1^, 75 or 60 kg P_2_O_5_ ha^–1^, and 150 kg K_2_O ha^–1^, respectively (Zheng et al., 2016). These fertilizer rates were calculated based on the common practices in the area. For all treatments, both coated controlled-release nitrogen and conventional nitrogen were used to provide 60 and 40% of the total applied nitrogen, respectively (Zheng et al., 2016; Qu et al., 2020). HA was applied at 45 kg ha^–1^.

On June 20, 2017, three seeds of maize (*Zea mays* L. cv. Zhengdan 958) were sown in each container. At the three-leaf stage, the seedlings were reduced to one. Agricultural management, such as pest and weed control, was performed as needed according to local practices. In 2018, the experiment was repeated using the same pots. Maize was planted on June 12, 2018, and harvested on September 26, 2018.

Maize ears were harvested after maturity on September 29, 2017, and September 26, 2018, respectively. To deactivate enzymes, kernels and plant samples were oven-dried at 105°C for 15 min, then dried at 65°C to a constant weight (Zheng et al., 2016; Gao et al., 2021). The biomass and yield of the maize were measured.

### Sampling Analyses

To learn the nutrient release pattern of CDAP, 10 g of CDAP was placed in a glass bottle containing 200 ml distilled water and incubated at 25^°^C. The solution in the bottle was sampled at days 1, 3, 5, 7, 10, 14, 28, 42, 56, 70, 84, 98, and 112 and analyzed for N and P concentrations according to the National Standard of the People’s Republic of China—Slow-Release Fertilizers (Liu et al., 2009). The functional groups of HA were identified with an FT-IR TENSOR analyzer (Bruker Co., Germany).

In 2017, at the growth stages of seedling (V3), six-leaf (V6), twelve-leaf (V12), and milk stages (R3) of soil samples were taken from 0 to 20 cm layer of each pot, air-dried, ground, and sieved to < 2 mm; plant height was measured from the soil surface to the top of the plant stem; the diameter of the maize stem was measured at the middle of the third node from the soil surface; The readings from the Soil Plant Analysis Development (SPAD) chlorophyll meter were taken between 09:00 and 11:00 a.m. (SPAD-502, Minolta, Japan). Soil available P was extracted with 0.5 M NaHCO_3_ (pH = 8.5) and quantified using an automatic chemical analyzer (Smartchem200, AMS, Italy). Soil NO_3_^–^-N and NH_4_^+^-N were extracted with 0.01 M CaCl_2_ (1:10 soil to water ratio) and measured with a continuous-flow injection analyzer (AA3-A001-02E, Bran-Luebbe, Germany; Houba et al., 1986; Dou et al., 2000). Soil available K was extracted with 1.0 M CH_3_COONH_4_ and determined using a flame photometer (Lu, 2000).

In 2018, at the V12 stage, the photosynthetic rates were determined between 09:00 and 11:00 a.m. using a LI-6400XT portable photosynthesis system (LI-Cor, Lincoln, NE, United States). Then, the fresh plant leaves, roots, and soil were sampled and frozen in liquid nitrogen for biochemical analysis. Contents of phosphoenolpyruvate carboxylase (PEPC), ADP-glucose pyrophosphorylase (AGPase), adenosine triphosphate (ATP) synthase, pyruvate phosphate dikinase (PPDK), auxin–indole-3-acetic acid (IAA), cytokinin (CTK), abscisic acid (ABA), and gibberellin (GA) of maize leaves were measured using the ELISA kit from Shanghai HengYuan Biological Technology Co., Ltd. (Shanghai, China) according to the manufacturer’s instructions. Acid phosphatase (AP) and alkaline phosphatase (ALP) activities of maize root and soil were determined using the ELISA kit.

Total P content in the plant was measured using the molybdenum-antimony method after digestion with H_2_SO_4_-H_2_O_2_ (Lu, 2000). For the P fertilization treatments, PUE was calculated as follows (Devkota et al., 2013):

PUE (%) = (maize P uptake– maize P uptake in Control)/total P from fertilizer × 100%

### Statistical Analyses

**FIGURE 1:**
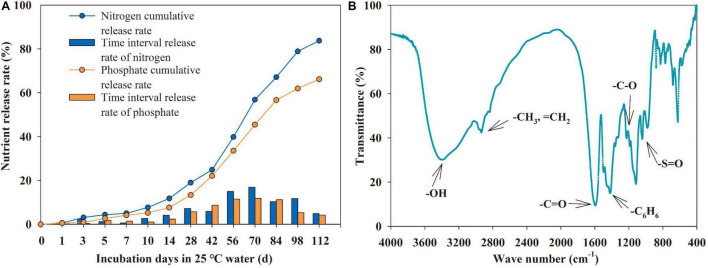
Release rates and accumulative release rates of nutrient from coated diammonium phosphate (CDAP) in 25^°^C water **(A)**; infrared spectrogram of humic acid (HA) **(B)**.

**FIGURE 2:**
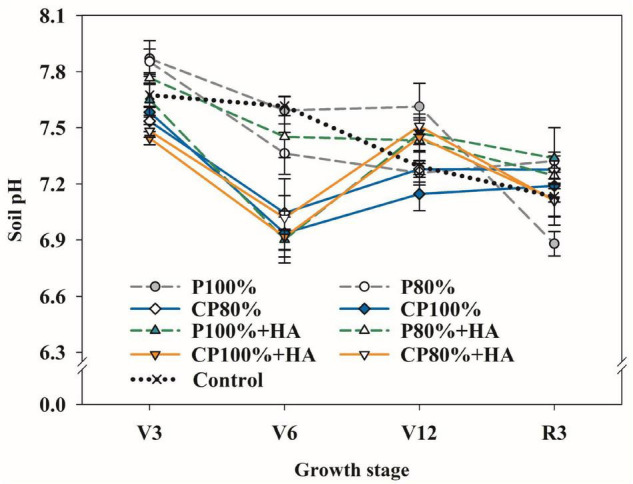
Changes of soil pH in different fertilization treatments. Control, no P fertilizer added; P 100%, diammonium phosphate (DAP) at 75 kg P_2_O_5_ ha^–1^; P80%, DAP at 60 kg P_2_O_5_ ha^–1^; CP 100%, coated DAP (CDAP) at 75 kg P_2_O_5_ ha^–1^; CP 80%, CDAP at 60 kg P_2_O_5_ ha^–1^; P 100% + HA, DAP at 75 kg P_2_O_5_ ha^–1^ and humic acid (HA); P 80% + HA, DAP at 60 kg P_2_O_5_ ha^–1^ and HA; CP 100% + HA, CDAP at 75 kg P_2_O_5_ ha^–1^ and HA; CP 80% + HA, CDAP at 60 kg P_2_O_5_ ha^–1^ and HA. V3, seedling stage; V6, six-leaf stage; V12, twelve-leaf stage; R3, milk stage.

**FIGURE 3:**
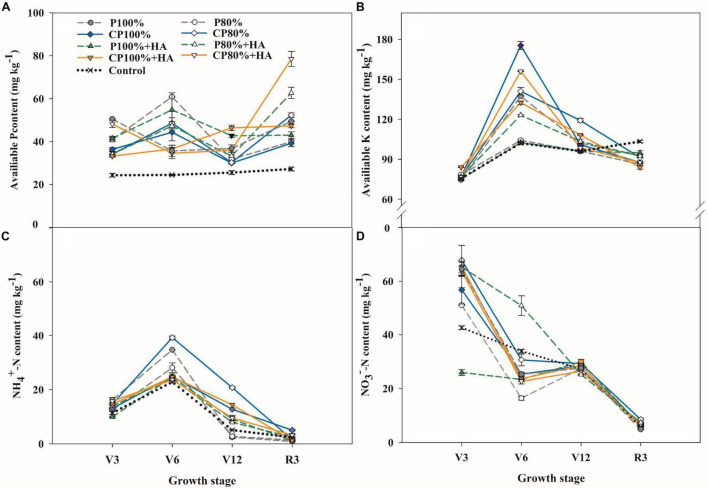
Changes of soil available P **(A)**, available K **(B)**, NH_4_^+^-N **(C)**, and NO_3_^–^-N **(D)** contents in different fertilization treatments. Control, no P fertilizer added; P 100%, diammonium phosphate (DAP) at 75 kg P_2_O_5_ ha^–1^; P80%, DAP at 60 kg P_2_O_5_ ha^–1^; CP 100%, coated DAP (CDAP) at 75 kg P_2_O_5_ ha^–1^; C P80%, CDAP at 60 kg P_2_O_5_ ha^–1^; P100%+HA, DAP at 75 kg P_2_O_5_ ha^–1^ and combined with humic acid (HA); P 80% + HA, DAP at 60 kg P_2_O_5_ ha^–1^ and combined with HA; CP 100% + HA, CDAP at 75 kg P_2_O_5_ ha^–1^ and combined with HA; CP 80% + HA, CDAP at 60 kg P_2_O_5_ ha^–1^ and combined with HA. V3, seedling stage; V6, six-leaf stage; V12, twelve-leaf stage; R3, milk stage.

**FIGURE 4:**
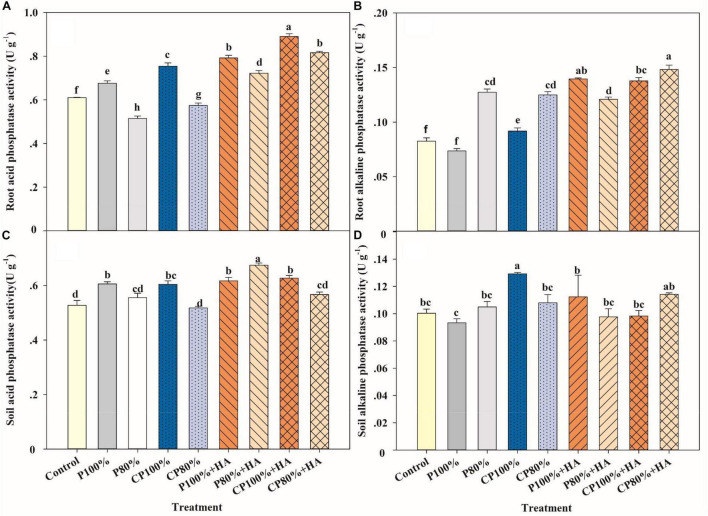
Activities of root acid phosphatase **(A)**, root alkaline phosphatase **(B)**, soil acid phosphatase **(C)**, and soil alkaline phosphatase **(D)** in different fertilization treatments at the twelve-leaf stage of maize. Control, no P fertilizer added; P 100%, diammonium phosphate (DAP) at 75 kg P_2_O_5_ ha^–1^; P80%, DAP at 60 kg P_2_O_5_ ha^–1^; CP 100%, coated DAP (CDAP) at 75 kg P_2_O_5_ ha^–1^; CP 80%, CDAP at 60 kg P_2_O_5_ ha^–1^; P 100% + HA, DAP at 75 kg P_2_O_5_ ha^–1^ and combined with humic acid (HA); P 80% + HA, DAP at 60 kg P_2_O_5_ ha^–1^ and combined with HA; CP 100% + HA, CDAP at 75 kg P_2_O_5_ ha^–1^ and combined with HA; CP 80% +HA, CDAP at 60 kg P_2_O_5_ ha^–1^ and combined with HA. Different letters above the bars indicate significant differences at *P* < 0.05 followed by Duncan’s multiple range test.

**FIGURE 5:**
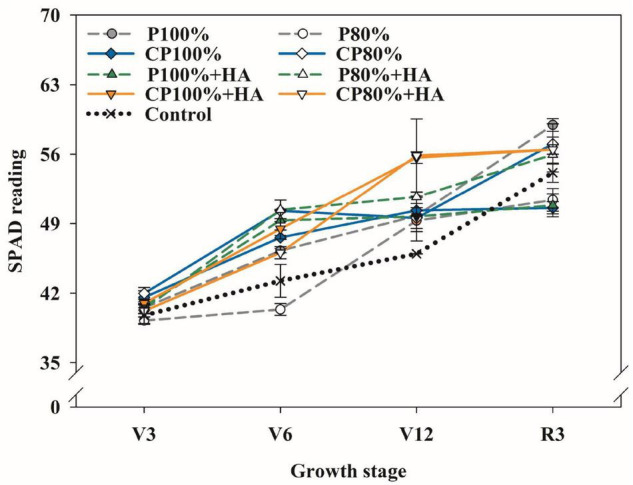
Changes in the reading of Soil Plant Analysis Development (SPAD) chlorophyll meter with maize growth in different fertilization treatments. Control, no P fertilizer added; P 100%, diammonium phosphate (DAP) at 75 kg P_2_O_5_ ha^–1^; P 80%, DAP at 60 kg P_2_O_5_ ha^–1^; CP 100%, coated DAP (CDAP) at 75 kg P_2_O_5_ ha^–1^; CP 80%, CDAP at 60 kg P_2_O_5_ ha^–1^; P 100% + HA, DAP at 75 kg P_2_O_5_ ha^–1^ and combined with humic acid (HA); P 80% +H A, DAP at 60 kg P_2_O_5_ ha^–1^ and combined with HA; CP 100% + HA, CDAP at 75 kg P_2_O_5_ ha^–1^ and combined with HA; CP 80% + HA, CDAP at 60 kg P_2_O_5_ ha^–1^ and combined with HA. V3, seedling stage; V6, six-leaf stage; V12, twelve-leaf stage; R3, milk stage.

**FIGURE 6:**
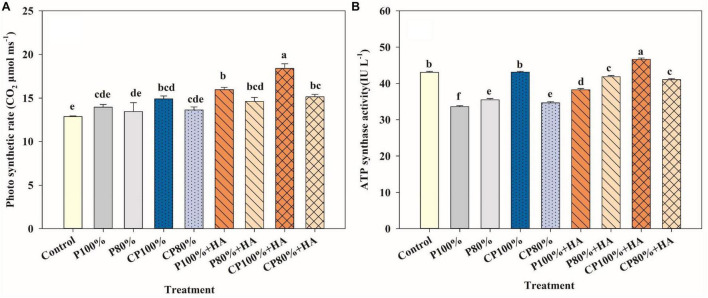
Photosynthetic rate **(A)** and ATP synthase activity **(B)** of maize in different fertilization treatments at the twelve-leaf stage. Control, no P fertilizer added; P 100%, diammonium phosphate (DAP) at 75 kg P_2_O_5_ ha^–1^; P80%, DAP at 60 kg P_2_O_5_ ha^–1^; CP 100%, coated DAP (CDAP) at 75 kg P_2_O_5_ ha^–1^; CP 80%, CDAP at 60 kg P_2_O_5_ ha^–1^; P 100% + HA, DAP at 75 kg P_2_O_5_ ha^–1^ and combined with humic acid (HA); P 80% + HA, DAP at 60 kg P_2_O_5_ ha^–1^ and combined with HA; CP 100% + HA, CDAP at 75 kg P_2_O_5_ ha^–1^ and combined with HA; CP 80% + HA, CDAP at 60 kg P_2_O_5_ ha^–1^ and combined with HA. Different letters above the bars indicate significant differences at *P* < 0.05 followed by Duncan’s multiple range test.

**FIGURE 7:**
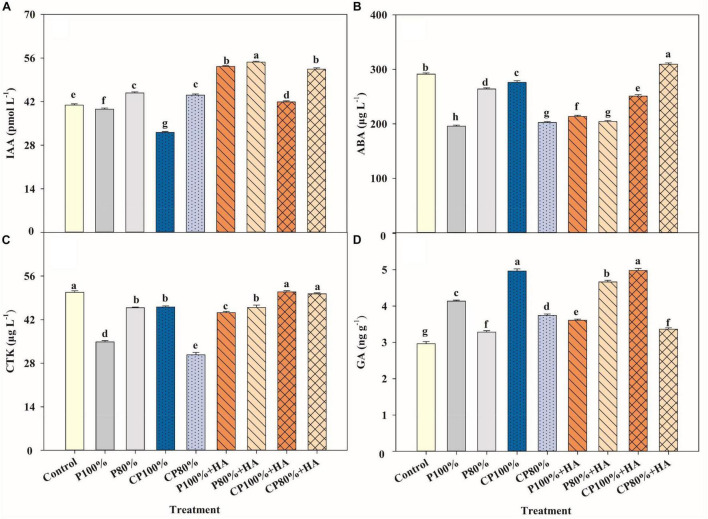
Auxin indole-3-acetic acid (IAA) **(A)**, abscisic acid (ABA) **(B)**, cytokinin (CTK) **(C)** and gibberellin GA **(D)** contents of maize leaves in different fertilization treatments at the twelve-leaf stage. Control, no P fertilizer added; P 100%, diammonium phosphate (DAP) at 75 kg P_2_O_5_ ha^–1^; P80%, DAP at 60 kg P_2_O_5_ ha^–1^; CP 100%, coated DAP (CDAP) at 75 kg P_2_O_5_ ha^–1^; CP8 0%, CDAP at 60 kg P_2_O_5_ ha^–1^; P 100% + HA, DAP at 75 kg P_2_O_5_ ha^–1^ and combined with humic acid (HA); P 80% + HA, DAP at 60 kg P_2_O_5_ ha^–1^ and combined with HA; CP 100% + HA, CDAP at 75 kg P_2_O_5_ ha^–1^ and combined with HA; CP 80% + HA, CDAP at 60 kg P_2_O_5_ ha^–1^ and combined with HA. Different letters above the bars indicate significant differences at *P* < 0.05 followed by Duncan’s multiple range test.

**FIGURE 8:**
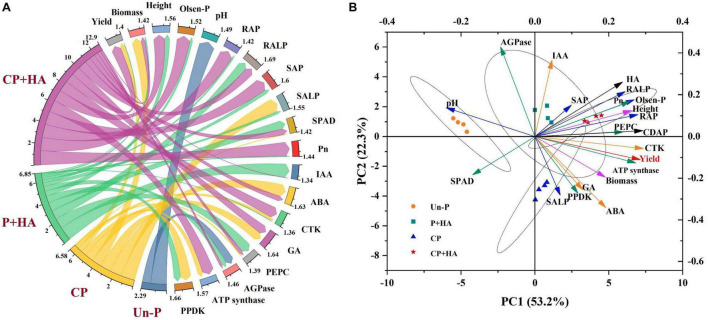
**(A)** Chord diagram for showing the effects of the different treatments on these indices. This plot links these treatments *via* ribbons to their associated indexes. **(B)** Principal component analysis shows the relationship among grain yield, growth parameters, and soil nutrient. Height, plant height; SAP, soil acid phosphatase; SALP, soil alkaline phosphatase; RAP, root acid phosphatase; RALP, root alkaline phosphatase; Pn, photosynthetic rate; PEPC, Phosphoenolpyruvate carboxylase; AGPase, ADP-glucose pyrophosphorylase; PPDK, pyruvate phosphate dikinase; IAA, indole-3-acetic acid auxin; CTK, cytokinin; ABA, abscisic acid; GA, gibberellin. Un-P, uncoated DAP treatments (P 100% and P 80% treatments); CP, coated DAP treatments (CP 100% and CP 80% treatments); P + HA, uncoated DAP combined with HA treatments (P 100% + HA and P 80% +HA treatments); CP + HA, coated DAP combined with HA treatments (CP 100% + HA and CP 80% + HA treatments).

**FIGURE 9:**
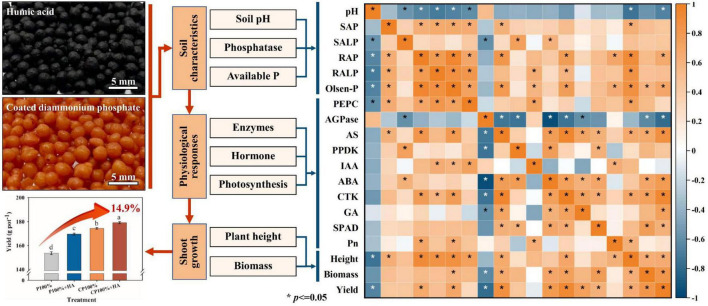
Correlation relations between maize yield and different indices. Spearman’s correlation between soil variables, growth parameters, and maize yields. Blue represents negative correlation and red represents positive correlation. The darker the color, the stronger the correlation, and vice versa (*P ≤ 0.05). Height, plant height; SAP, soil acid phosphatase; SALP, soil alkaline phosphatase; RAP, root acid phosphatase; RALP, root alkaline phosphatase; Pn, photosynthetic rate; PEPC, Phosphoenolpyruvate carboxylase; AGPase, ADP-glucose pyrophosphorylase; PPDK, pyruvate phosphate dikinase; IAA, indole-3-acetic acid auxin; CTK, cytokinin; ABA, abscisic acid; GA, gibberellin.

Data were collected and analyzed with Microsoft Excel 2010, and figures were generated using SigmaPlot software (Version 12.5, MMIV, Systat Software Inc., San Jose, CA, United States; Figures 1–7) and Origin software (Version 2021b, OriginLab Corporation, MA, United States; Figure 8 and Spearman’s correlation analysis in Figure 9). Analysis of variance technique (one-way ANOVA) with mean separation using Duncan’s test (*P* < 0.05) was performed with IBM SPSS Statistics 22 (SPSS Inc., IL, United States).

Origin software was used to determine the determinants on the application of different fertilizations in impacting maize yield and PUE using the chord diagram, principal component analysis, and Spearman’s correlation analysis (Hou et al., 2019; Li R. C. et al., 2021). With the exception of the control treatment, data were collected and divided into four categories: (1) uncoated DAP treatments (Un-P; the mean value of P 100% and P 80% treatments); (2) coated DAP treatments (CP; the mean value of CP 100% and CP 80% treatments); (3) uncoated DAP combined with HA treatments (P + HA; the mean value of P 100% + HA and P 80% + HA treatments); (4) coated DAP combined with HA treatments (CP + HA; the mean value of CP 100 % + HA and CP 80% +HA treatments).

## Results

### Nutrient Release Pattern of Coated Diammonium Phosphate and Functional Groups of Humic Acid

Under laboratory conditions in water (25°C), nutrient release from CDAP followed a linear pattern over time (Figure 1A). By day 112, the cumulative release rates of P and N from CDAP reached 66.1 and 83.7%, respectively. The release of P and N was steady during the first 10 days (5.2 and 7.7% released, respectively), accelerated during the days 10–28 (8.1, 11.3% released, respectively), and slowed down afterward. However, the final cumulative release rates of P and N were different.

The FT-IR spectrum of HA displayed several characteristic peaks (Figure 1B). The peaks at 1,226 and 1,188 cm^–1^ are due to the existence of the C-O group. The peak at 1,117 cm^–1^ is be attributed to the C-H stretching of the benzene ring or C-O stretching. The characteristic peak at 3,396 cm^–1^ corresponds to the stretching vibration of O-H in an aromatic ring (Sarlaki et al., 2021).

### Soil pH and Contents of Available Nutrients

The dominant form of orthophosphate ion present in the soil is pH dependent. At the V3 stage, the soil pH in CP 100%, P 100% + HA, and CP 100% +HA was 0.29, 0.22, and 0.10 units, respectively, lower than that in P 100% (Figure 2). The soil pH in CP 80%, P 80% + HA, and CP 80% + HA was significantly reduced by 0.33, 0.43, and 0.39 units, respectively, while that in P 80% was only reduced by 0.02 units compared to P1 00%. The P 100% treatment resulted in the highest soil pH at the V12 stage. From the V12 to the R3 stage, the largest decrease in soil pH (0.7 units) occurred in P 100%.

Soil available P content was affected by the P fertilizers and fertilizer application rate (Figure 3A). The control treatment displayed the lowest soil available P content at the V3, V6, V12, and R3 growth stages. The application of CDAP significantly increased the soil available P content during the late growth stages of maize. At the V12 stage, soil available P content in CP 100%, P 100% + HA, and CP 100% + HA was 1.3, 8.0, and 16.7%, respectively, higher than that in P 100%. At the lower P application level (60 kg P_2_O_5_ ha^–1^), the CP 80%, P 80% + HA, and CP 80% + HA treatments significantly increased the soil available P content by 38.8, 50.6, and 19.5%, respectively, compared to the P 100% treatment. When coated with polyurethane or applied together with HA, the DAP increased the soil available P content at the late growth stages of maize. At the V12 and R3 stages, soil available P content in CP 100%, P 100% + HA, and CP 100% + HA was 18.1, 35.7, and 61.8%, respectively, higher than that in P100%. The average soil available P content in CP 100% + HA was 47.8 mg kg^–1^, significantly higher than that in CP 100% by 9.5%.

Soil inorganic N (NO_3_^–^-N and NH_4_^+^-N) content (Figures 3C,D) was high at the early growth stages and decreased at the late growth stages of maize in all treatments. At the V6, V12, and R3 stages, soil inorganic N content in the treatments with combined application of P fertilizer (CDAP or DAP) and HA was higher than that in the treatments applied with DAP only. At the V12 stage, the inorganic N content in CP 100% and P 100% + HA was higher than that in P 100%. The highest inorganic N content was found in CP 80% while the lowest was in P 100% at the V12 stage. In the treatments with P fertilization, soil available K content was low at the V3 stage, which increased rapidly to the highest value at the V6 stage and then decreased afterward (Figure 3B). At the V6 stage, soil available K content in CP 100% and CP 80% was 27.7 and 2.7%, respectively, higher than that in P 100%.

### Acid Phosphatase and Alkaline Phosphatase Activities of Root and Soil

Phosphatase is a very important hydrolase that is ubiquitous in plants and soil. The root AP activity was increased by 11.6 and 17.2%, while the root ALP activity was increased by 24.7 and 89.5% in CP 100% and P 100% + HA, respectively, compared to P 100% (Figures 4A,B). The CP 100% + HA treatment increased the activities of root AP and ALP by 18.1 and 50.1%, respectively, compared to CP 100%. The root AP and ALP activities in CP 80% + HA were higher than those in CP 100%, though the P application rate was 20% lower in CP 80% + HA.

The highest soil AP activity was found in P 80% + HA, while the highest soil ALP activity was found in CP 100% (Figures 4C,D). There were no significant differences in soil AP activity between the treatments with 75 kg P_2_O_5_ ha^–1^. There were no significant differences in soil ALP activity between P 100% + HA and P 100%. The combined application of CDAP and HA did not have a clear effect on soil AP and ALP activities.

### Soil Plant Analysis Development Value, Photosynthetic Rate, and Photosynthesis Enzyme Activities

The readings from the SPAD value were employed to indicate the chlorophyll content of leaves, and the SPAD-502 chlorophyll meters were used to estimate it. All treatments exhibited an increasing trend in SPAD values over time (Figure 5). At the V12 stage, the SPAD value in the treatments with the application of CDAP, the treatments with the combined application of DAP and HA, and the treatments with the combined application of CDAP and HA were 2.1–8.4%, 4.9–7.9%, and 8.2–12.9%, respectively, higher than that in the treatments with the application of DAP at the same P rate. In addition, the SPAD value was significantly increased in CP 80%, P 80% + HA, and CP 80% + HA by 9.5, 12.5, and 21.1%, respectively, compared to P 100%, though 20% less P was applied in these treatments. The application of HA increased the leaf SPAD value of maize.

Photosynthesis is a fundamental physiological process of maize that uses light energy to accumulate organic matter. The combined application of CDAP and HA enhanced photosynthesis at the V12 stage, a vital growth stage of maize (Figure 6). The photosynthetic rate in CP 100% + HA was the highest of all the treatments. For the treatments with 75 kg P_2_O_5_ ha^–1^, the photosynthetic rate was in the order of CP 100% + HA > P 100% + HA > CP 100%. Of the treatments with 60 kg P_2_O_5_ ha^–1^, P 80% + HA had the highest photosynthetic rate. Photosynthesis, a process that involves many enzymes, is strongly affected by the orthophosphate concentrations in cytosol and chloroplast. The different P treatments showed different effects on the activities of photosynthesis-related enzymes (Figure 6 and Supplementary Figure 1). Of all the treatments, P 80% had the lowest PEPC and PPDK activities (Supplementary Figure 1). The addition of HA significantly increased the activities of PEPC, ATP synthase, and PPDK.

### Endogenous Hormones in Maize Leaf

Endogenous hormones serve a critical role in plant growth and development, even at very low levels. Figure 7 showed the contents of IAA, ABA, CTK, and GA in maize leaves during the V12 stage. Compared to P 100%, the CTK and GA contents in CP 100% were increased by 32.4 and 21.1%, respectively. The P 100% + HA treatment increased IAA content by 34.6% and CTK content by 27.2%. The CP 100% + HA treatment increased IAA, CTK, and GA contents by 5.8, 46.4, and 21.5%, respectively. Moreover, the IAA and CTK contents were 30.5 and 10.6%, respectively, higher in CP 100% + HA and 63.3 and 9.2%, respectively, higher in CP 80% + HA than those in CP 100%.

### Maize Plant Height and Stem Diameter

Changes in plant height and stem diameter at different maize growth stages are presented in Table 1. The maize plants in the control treatment were the shortest with the slenderest stems at the V3, V6, V12, and R3 stages. Compared to P 100%, the treatments with CP 100%, P 100% + HA, and CP 100% + HA increased the plant height by 3.7, 7.3, and 9.9%, respectively, while CP 80%, P 80% + HA, and CP 80% + HA increased the plant height by 7.8, 6.6, and 10.1%, respectively. At the V3 stage, CP 100%, P 100% + HA, and CP 100% + HA increased the diameter of the maize stem by 10.4–20.0%, 10.1–15.1%, and 7.8–12.6%, respectively, compared to P 100%.

**TABLE 1:** Plant height and stem diameter of maize in different fertilization treatments.

Treatment^a^	Plant height (cm)				Plant stem diameter (mm)			
	V3^b^	V6	V12	R3	V3	V6	V12	R3
Control	54.3a^c^	95.3a	210.0a	223.1a	10.0d	20.3b	21.9a	25.5a
P100%	60.5a	112.6a	202.5a	224.0a	12.8abc	25.9a	25.5a	28.2a
P80%	57.9a	111.9a	234.8a	233.4a	11.2cd	22.2ab	23.2a	26.6a
CP100%	62.9a	116.0a	234.0a	232.3a	14.1a	24.7ab	24.9a	27.5a
CP80%	61.8a	117.0a	241.0a	241.5a	13.4ab	24.6ab	25.1a	27.8a
P100%+HA	63.4a	120.9a	239.8a	240.4a	14.1a	25.0ab	24.2a	27.8a
P80%+HA	62.4a	111.3a	236.3a	238.8a	12.9abc	22.1ab	22.2a	26.4a
CP100%+HA	59.3a	114.1a	245.5a	246.3a	14.4a	23.8ab	24.4a	26.4a
CP80%+HA	59.2a	114.5a	232.0a	246.6a	12.1bc	23.7ab	25.2a	26.0a

*^a^Control, no P fertilizer added; P 100%, diammonium phosphate (DAP) at 75 kg P_2_O_5_ ha^–1^; P 80%, DAP at 60 kg P_2_O_5_ ha^–1^; CP 100%, coated DAP (CDAP) at 75 kg P_2_O_5_ ha^–1^; CP 80%, CDAP at 60 kg P_2_O_5_ ha^–1^; P100%+HA, DAP at 75 kg P_2_O_5_ ha^–1^ and combined with humic acid (HA); P 80% + HA, DAP at 60 kg P_2_O_5_ ha^–1^ and combined with HA; CP 100% + HA, CDAP at 75 kg P_2_O_5_ ha^–1^ and combined with HA; CP 80% + HA, CDAP at 60 kg P_2_O_5_ ha^–1^ and combined with HA.*

*^b^Growth stages: V3, seedling stage; V6, six-leaf stage; V12, twelve-leaf stage; R3, milk stage.*

*^c^Means within each column followed by the same letters were not significantly different based on one-way ANOVA followed by Duncan’s test (P < 0.05).*

### Maize Yield, P-Use Efficiency, and Net Profit of Maize Production

In both 2017 and 2018, the maize yield of the control treatment was significantly lower than that of the other treatments (Table 2). Compared to P 100%, the 2-year average grain yield in CP 100% and P 100% + HA was significantly higher by 13.5 and 10.3%, respectively, while that in CP 80% and P 80% + HA, the grain yield was higher by 11.0 and 10.0%, respectively. The grain yield in CP 100% + HA was 17.2 and 16.0% higher than that in P 100% in 2017 and 2018, respectively. In 2018, the highest yield was obtained in CP 100% + HA, which was 4.2 and 4.7% higher than that in CP 100% and P 100% + HA, respectively. The PUE of CDAP or DAP combined with HA was significantly higher than that of DAP (Table 2). The 2-year average PUE in CP 100%, P 100% + HA, and CP 100% + HA was 24.4, 13.8, and 27.8 percentage points, respectively, higher than that in P 100%. The CP 80%, P 80% + HA, and CP 80% + HA treatments achieved a higher average of PUE than the P 100% treatment by 26.4, 20.7, and 27.4 percentage points, respectively.

**TABLE 2:** Maize yield and phosphorus use efficiency (PUE) in different fertilization treatments.

Treatment^a^	Kernel (ear^–1^)	Plant biomass (g pot^–1^)	Yield (g pot^–1^)	Yield change vs. P100% (%)	PUE (%)	PUE change vs. P100%
2017						
Control	487b^b^	267.0d	130.3d	−9.1	–	–
P100%	543ab	301.2c	143.4c	0.0	16.7f	–
P80%	488b	304.0c	140.8c	−1.8	17.2f	0.5
CP100%	556ab	329.2a	166.2a	15.9	37.2c	20.5
CP80%	492b	318.1b	159.4b	11.1	39.3c	22.6
P100%+HA	600a	308.1c	159.1b	10.9	25.2e	8.5
P80%+HA	517b	324.2ab	158.9b	10.8	33.0d	16.3
CP100%+HA	523b	322.9ab	168.1a	17.2	40.3b	23.5
CP80%+HA	515b	319.9ab	161.2b	12.4	42.2a	25.5
2018						
Control	508c	319.5e	149.6e	−8.8	–	–
P100%	585abc	334.6d	164.0d	0.0	15.5f	–
P80%	573bc	347.4c	164.5d	0.3	16.6f	1.1
CP100%	650ab	372.8a	182.6bc	11.3	43.8c	28.3
CP80%	651ab	359.1bc	181.8c	10.9	45.7b	30.3
P100%+HA	629ab	376.0a	180.0c	9.7	34.6e	19.1
P80%+HA	603ab	351.7c	179.3c	9.3	40.6d	25.1
CP100%+HA	669a	382.9a	190.3a	16.0	47.5b	32.1
CP80%+HA	650ab	370.7ab	186.7ab	13.8	44.9a	29.4

*^a^Control, no P fertilizer added; P 100%, diammonium phosphate (DAP) at 75 kg P_2_O_5_ ha^–1^; P 80%, DAP at 60 kg P_2_O_5_ ha^–1^; CP 100%, coated DAP (CDAP) at 75 kg P_2_O_5_ ha^–1^; CP 80%, CDAP at 60 kg P_2_O_5_ ha^–1^; P 100% + HA, DAP at 75 kg P_2_O_5_ ha^–1^ and combined with humic acid (HA); P 80% + HA, DAP at 60 kg P_2_O_5_ ha^–1^ and combined with HA; CP 100% + HA, CDAP at 75 kg P_2_O_5_ ha^–1^ and combined with HA; CP 80% + HA, CDAP at 60 kg P_2_O_5_ ha^–1^ and combined with HA.*

*^b^Means within each column in each year followed by the same letters were not significantly different based on one-way ANOVA followed by Duncan’s test (P < 0.05).*

When DAP was used in combination with HA, or when CDAP was applied alone or in combination with HA, the maize production cost was higher than when DAP was applied alone (Table 3). However, the application of CDAP, alone or together with HA, generally increased the maize grain yield. Of all treatments, CP 100% achieved the highest average net income. Compared to P 100%, the average net income of CP 100% and CP 80% was increased by 483.1 and 440.8 USD ha^–1^, respectively, while that of P 100% + HA and P 80% + HA was increased by 287.1 and 321.5 USD ha^–1^, respectively. The highest income was achieved by the CP 100% + HA treatment, which was 485.6 and 198.5 USD ha^–1^ greater than that by the P 100% and P 100%+HA treatments, respectively.

**TABLE 3:** Cost and net profits of maize production in different fertilization treatments.

Treatment^b^	Cost^a^ (USD ha^–1^ year^–1^)		Total income (USD ha^–1^)		Net income (USD ha^–1^)			
	P fertilizer	Labor	2017	2018	2017	2018	Average	Change vs. P100%
Control	405.4	72.5	3403.5d*^c^*	3906.7e	2925.6e	3428.8d	3177.2e	−4.0
P100%	633.2	72.5	3744.8c	4282.7d	3039.1d	3577.0c	3308.0d	–
P80%	590.0	72.5	3676.2c	4295.5d	3013.7d	3633.0c	3323.3d	0.5
CP100%	690.4	72.5	4339.9a	4768.5bc	3576.8a	4005.4a	3791.1a	14.6
CP80%	633.8	72.5	4162.1b	4748.6c	3455.6b	4042.1a	3748.8ab	13.3
P100%+HA	759.4	72.5	4154.6b	4699.8c	3322.5c	3867.6b	3595.1c	8.7
P80%+HA	712.9	72.5	4149.4b	4680.9c	3363.7c	3895.3b	3629.5c	9.7
CP100%+HA	813.1	72.5	4390.0a	4969.0a	3504.2b	4083.1a	3793.6a	14.7
CP80%+HA	756.6	72.5	4209.7b	4875.5ab	3380.4c	4046.2a	3713.3b	12.3

*^a^Based on the current mean market price. Data in the table were calculated based on the current mean market price; maize, 313.4 USD t^–1^; CDAP (coated diammonium phosphate), 557.4 USD t^–1^; DAP (diammonium phosphate), 447.8 USD t^–1^; Urea, 238.8 USD t^–1^; Controlled-release urea, 348.4 USD t^–1^; Potassium chloride, 373.1 USD t^–1^; humic acid (HA), 302 USD t^–1^; Labor cost for seeding, field management, and harvest, 72.5 USD ha^–1^; and other costs including those for machinery, irrigation, pesticides, insecticides, seeds, and other materials and expenses, 1320.5 USD ha^–1^.*

*^b^Control, no P fertilizer added; P 100%, DAP at 75 kg P_2_O_5_ ha^–1^; P 80%, DAP at 60 kg P_2_O_5_ ha^–1^; CP 100%, CDAP at 75 kg P_2_O_5_ ha^–1^; CP 80%, CDAP at 60 kg P_2_O_5_ ha^–1^; P 100% + HA, DAP at 75 kg P_2_O_5_ ha^–1^ and combined with HA; P 80% + HA, DAP at 60 kg P_2_O_5_ ha^–1^ and combined with HA; CP 100% +HA, CDAP at 75 kg P_2_O_5_ ha^–1^ and combined with HA; CP 80% + HA, CDAP at 60 kg P_2_O_5_ ha^–1^ and combined with HA.*

*^c^Means within each column followed by the same letters were not significantly different based on one-way ANOVA followed by Duncan’s test (P < 0.05).*

### Correlation Analysis

Correlation analysis showed that there was a positive effect with the application of CDAP in combination with HA on yield, biomass, height, available P content, soil AP, soil ALP, root AP, root ALP, IAA, ABA, CTK, GA, PEPC, AGPase, and ATP synthase (Figure 8A). In addition, it was obvious that the contribution of the application of CDAP combined with HA respond to these indices than other treatments, including the combination of DAP and HA and application of CDAP and DAP, respectively. Principal component analysis revealed that the 21 parameters were divided into PC 1 (53.6%) and PC 2 (22.3%). PC 1 and PC 2 explained 53.2% of the differences among the 21 indicators (Figure 8B). In addition, pH and AGPase were distributed in the second quadrant, and they had a negative relationship with the other parameters mainly distributed in the first and fourth quadrants.

## Discussion

### Effects of Coated Diammonium Phosphate on P Supply Intensity in the Soil Solution and Its Availability

It is important to balance fertilizer input and crop uptake in high-yielding maize production (Shoji et al., 2001; Geng et al., 2015). The nutrient release curve of CDAP (Figure 1A) is similar to the sigmoidal nutrient uptake curve of maize (Bender et al., 2013). Lu et al. (2020) reported similar results pertaining to the nutrient release characteristics of CDAP that match with the nutrient uptake requirement of maize much better than the conventional P fertilizer. Based on soil available P changes during maize growth (Figure 3A), it is speculated that CDAP had a longer P-release period in the pot experiment than in the laboratory incubation (Figure 1A). First, the coating of CDAP was done by a polyurethane block copolymer containing soft and hard segments, with swelling properties for gradual nutrient release (Hou et al., 2015; Lu et al., 2016). Second, the nutrient release from coated fertilizers is greatly affected by humidity and temperature (Yang et al., 2011). Although soil temperature (26.1°C on average) during maize growth in the pot experiment was closer to the incubation temperature in the laboratory, soil moisture was low compared with the moisture condition in laboratory incubation (Li et al., 2020), which might have greatly slowed down the P release. Our findings were in line with those of Zheng et al. (2016), who discovered changes in nutrient release characteristics between field soils and laboratory soils.

Sufficient nutrient supply is a requirement for high crop yield. In this study, the maize yield in CP 100% and CP 80% was increased by 13.5 and 11.0%, respectively, compared to P 100% (Table 1). The coating of CDAP not only separates DAP from direct contact with the soil, thus preventing P from fixation by the soil *via* sorption, complexation, and precipitation (Roberts and Johnston, 2015) but also stops DAP from rapid dissolution and loss of surface runoff and subsurface flow (Hively et al., 2006; Holman et al., 2008). The V12 stage is a highly P-demanding growth stage of maize, during which a large amount of P is needed for grain development in the later stages (Bender et al., 2013). The application of CDAP increased the activity of phosphatase, AP in particular, in the root by 11.5–24.7% at the V12 stage (Figure 4), leading to higher P availability due to more organic P being hydrolyzed.

Additionally, the application of conventional P fertilizer (i.e., DAP) resulted in a higher soil available P content at the V3 stage (Figure 2), which would lead to a higher P content in the maize plant (Bender et al., 2013). However, high available P would inhibit the synthesis and the activity of phosphatase and in turn, would slow down the decomposition of protein-phytic acid-mineral element complex. Consequently, the availability of mineral elements would be decreased (Sarah et al., 2018). As CDAP released P into the soil solution at a rate that matches the maize demand, maize growth was not limited by P, resulting in a high grain yield.

### Effects of Humic Acid on the P Availability and Maize Growth

Humic acid, as a P activator, accelerated P transformation into bioavailable forms. On the one hand, soil pH was lower in the treatments with combined application of HA and DAP than in the treatments with an application of DAP alone at the V3 stage. The presence of HA increases the altering of the root exudate profile and it also enhances the release of oxalate and citrate from maize roots, compared to maize not treated with HA (Canellas et al., 2008; Rosa et al., 2018; Ma et al., 2021). Additionally, H^+^ is produced during HA decomposition (Hue, 1991), leading to an available P increase in calcareous soils with the dissolution of insoluble P compounds. On the other hand, the adsorption of HA generates a repulsive negative electric potential on the adsorption plane and a steric hindrance on the mineral surface, further inhibiting P binding on the soil surface (Wang, 2016). Additionally, HA complexes with Ca^2+^, Fe^3+^, and Al^3+^, reduce P precipitation with these cations. This was associated with the rich functional groups in HA, such as O-H in aromatic rings, C-O, and C-H in benzene rings (Figure 1B; Sarlaki et al., 2021). Furthermore, the application of HA increased the phosphatase (i.e., soil AP and root AP) activity (Figure 4) and in turn the amount of P released from the soil solid phase or from P fertilizers (Nardi et al., 2017; Zhu et al., 2018), leading to an increase in the available P content (Figure 3A). Therefore, HA protected P fertilizer to avoid wasting and improved the P availability in soil.

In addition to the P availability in soil, the HA use increased the growth of maize. As a biostimulant, HA possesses aliphatic and aromatic structures with various functional groups (mainly oxygen-containing). Its phenolic and quinone groups interact with enzymes in plant cell and stimulate plant metabolism, thereby promoting growth and improving crop yield (Fan et al., 2015). In this study, the photosynthetic rate of maize leaves at the key growth stage was enhanced by the application of HA (Figure 6). The application of HA improved photosynthesis by increasing the activities of PEPC, ATP synthase, and PPDK (Figure 6 and Supplementary Figure 1) and influenced the important metabolic pathways in photosynthesis, such as photosynthetic carbon assimilation, oxidative phosphorylation, and photosynthetic phosphorylation (Gao et al., 2017; Nardi et al., 2017).

The endogenous hormone plays a key role in regulating plant growth and developmental processes as well as in regulating plant responses to the external environment (Fahad et al., 2015a,b). The P 100% + HA treatment increased the IAA, ABA, and CTK contents in maize leaves by 34.6, 9.1, and 27.2%, respectively, compared to the P 100% treatment at the V12 stage. These results are in confirmation with the reports by Nardi et al. (2017). Nardi et al. (2002) suggested that humic-like substances behave as signaling molecules in the rhizosphere, eliciting the production of phytohormones. Moreover, HA stimulates the expression of *IAA5* and *IAA19*, two early auxin-responsive genes (Nardi et al., 2002). These phytohormones can act either locally (at the site of their synthesis) or transported to some other sites within the maize plant body to mediate growth and development responses of both under ambient and stressful conditions (Peleg and Blumwald, 2011; Fahad et al., 2015a). Furthermore, IAA and HA can enhance the synthesis and activity of plasma membrane H^+^-ATPase, an enzyme that converts energy for transmembrane transportation of nutrients including P, then energizes secondary ion transporters, and promotes the nutrient uptake of maize (Zandonadi et al., 2010).

### Interactive Effects of Coated Diammonium Phosphate and Humic Acid

The interactive effect of CDAP and HA improved maize grain yield and PUE (Figure 9). The CDAP synchronized the P supply with plant demand. The HA increased the photosynthetic rate of maize to accumulate organic matter. Additionally, the functional groups of HA, such as –COOH and –OH (Figure 1B), adsorb to the soil surface and react with soil minerals, influencing metal speciation and solubility and reducing P fixation (Nardi et al., 2017; Sarlaki et al., 2021), which is conducive to meeting the P demand of maize in the early growth stage. Furthermore, some studies have shown that HA was capable of promoting root growth and modifying root architecture (Olaetxea et al., 2018) as well as modifying the gene expression of the main high-affinity root transporters of phosphate to increase the phosphate root uptake (Jindo et al., 2016; Olaetxea et al., 2018). Therefore, the combination of CDAP and HA was able to improve the overall P nutrition in maize plants. Besides, the contents of IAA and CTK in maize leaves were improved when treated with CDAP combined with HA. Meanwhile, the ATP synthase activity and the photosynthetic rate were improved (Figure 9) leading to better crop growth and grain development.

The interactive effect of CDAP and HA on maize yield was not significant in 2017. This may be because maize is a highly P-efficient crop and not very sensitive to P levels in soil (Ciarelli et al., 1998; Ci et al., 2012). However, the yield in CP 100% + HA was significantly higher than that in CP 100% in 2018. This is because the combination of CDAP and HA exerts beneficial effects on plant growth by improving the soil structure, fertility, and quality (Calvo et al., 2014; Raiesi, 2021; Xu et al., 2021), and two consecutive years of application eventually resulted in significant changes. The combination of CDAP and HA provides an HA-incorporated enhanced-efficiency P fertilizer for environmentally friendly fertilization (Figure 9), and future research should include P-inefficient crops (e.g., wheat).

## Conclusion

The application of CDAP combined with HA increased the soil available P content, improved the root acid phosphatase activity, ATP synthase activity, and cytokinin content, increased the photosynthetic rate, and plant height, and eventually increased the maize grain yield and PUE. When P was applied at 75 kg P_2_O_5_ ha^–1^, higher maize grain yield was obtained by CDAP-HA combination than by CDAP alone or DAP-HA combination, and in the second year of cultivation, these differences became bigger and significant. Even at 60 kg P_2_O_5_ ha^–1^, the combined application of CDAP and HA presented a higher grain yield and PUE in the second year than the conventional fertilization. Overall, the combined application of CDAP and HA is of significance in improving PUE, reducing P loss to the environment, ensuring food security, realizing sustainable utilization of land and fertilizer resources, and increasing the economic return of crops. Future studies should be conducted in different regions with various soil types and crops to develop an effective strategy of fertilization with controlled-release fertilizers and biostimulants (e.g., HA).

## Data Availability Statement

The original contributions presented in the study are included in the article/Supplementary Material, further inquiries can be directed to the corresponding author/s.

## Author Contributions

QC, ZGL, ZW, and MZ contributed to the conception and design of the study. QC, ZQ, ZL, ZZ, and GM organized the database. QC, YW, LW, and FF contributed to investigating and obtaining the resources. QC, ZGL, ZW, and MZ wrote sections of the manuscript. QC wrote the first draft of the manuscript. All authors contributed to the article and approved the submitted version.

## Conflict of Interest

YW and LW were employed by the company Xinyangfeng Agricultural Technology Co., Ltd. The remaining authors declare that the research was conducted in the absence of any commercial or financial relationships that could be construed as a potential conflict of interest.

## Publisher’s Note

All claims expressed in this article are solely those of the authors and do not necessarily represent those of their affiliated organizations, or those of the publisher, the editors and the reviewers. Any product that may be evaluated in this article, or claim that may be made by its manufacturer, is not guaranteed or endorsed by the publisher.
