# Supplementary material for: Coated Diammonium Phosphate Combined With Humic Acid Improves Soil Phosphorus Availability and Photosynthesis and the Yield of Maize
Source: Front Plant Sci. 2021 Dec 16;12:759929. doi: 10.3389/fpls.2021.759929 (PMC8716685; doi:10.3389/fpls.2021.759929)
Supplement: Supplementary file 1 [file Data_Sheet_1.docx]

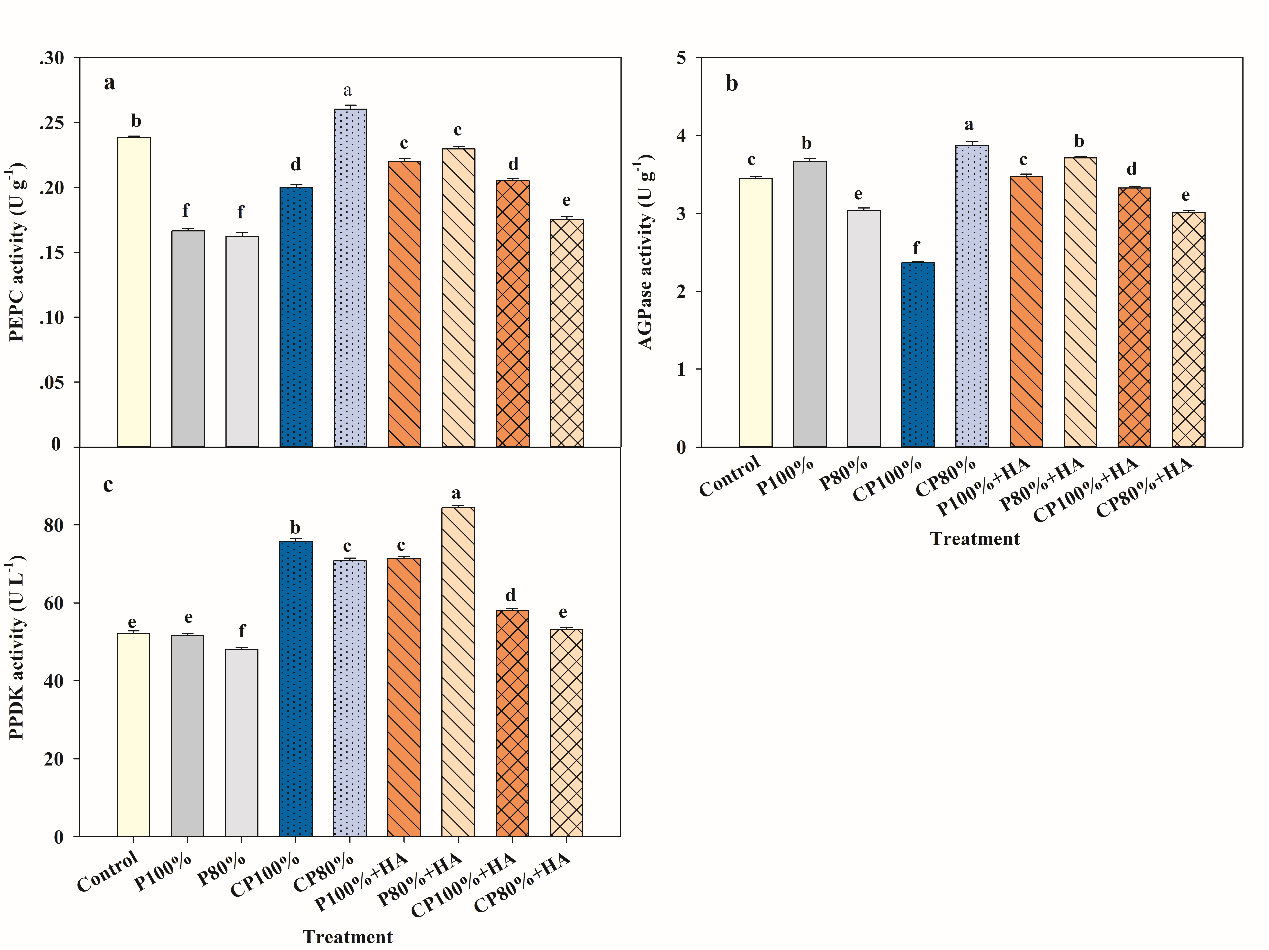


**FIGURE S1** Phosphoenolpyruvate carboxylase (PEPC) activity(a), ADP-glucose pyrophosphorylase (AGPase) activity (b), and pyruvate phosphate dikinase (PPDK) activity (c) of maize in different fertilization treatments at the twelve-leaf stage. Control: no P fertilizer added; P100%: diammonium phosphate (DAP) at 75 kg P_2_O_5_ ha^-1^; P80%: DAP at 60 kg P_2_O_5_ ha^-1^; CP100%: coated DAP (CDAP) at 75 kg P_2_O_5_ ha^-1^; CP80%: CDAP at 60 kg P_2_O_5_ ha^-1^; P100%+HA: DAP at 75 kg P_2_O_5_ ha^-1^ and combined with humic acid (HA); P80%+HA: DAP at 60 kg P_2_O_5_ ha^-1^ and combined with HA; CP100%+HA: CDAP at 75 kg P_2_O_5_ ha^-1^ and combined with HA; CP80%+HA: CDAP at 60 kg P_2_O_5_ ha^-1^ and combined with HA.
